# Associations between general self-efficacy and health-related quality of life among 12-13-year-old school children: a cross-sectional survey

**DOI:** 10.1186/1477-7525-7-85

**Published:** 2009-09-23

**Authors:** Lisbeth Gravdal Kvarme, Kristin Haraldstad, Sølvi Helseth, Ragnhild Sørum, Gerd Karin Natvig

**Affiliations:** 1 Diakonova University College, Linstowsgate 5, N-0166 Oslo, Norway; 2 Oslo University College, Pilestredet 46, N-0167 Oslo, Norway; 3 Cancer Registry of Norway, Postboks 5313 Majorstuen, N-0340 Oslo, Norway; 4 Department of Public Health and Primary Care, University of Bergen, Kalfarveien 31, N-5018 Bergen, Norway

## Abstract

**Background:**

While research on school children's health has mainly focused on risk factors and illness, few studies have examined aspects of health promotion. Thus, this study focuses on health promotional factors including general self-efficacy (GSE) and health-related quality of life (HRQOL). GSE refers to a global confidence in coping ability across a wide range of demanding situations, and is related to health. The purpose of this study was to examine associations between GSE and HRQOL, and associations between HRQOL and socio-demographic characteristics. Knowledge of these associations in healthy school children is currently lacking.

**Methods:**

During 2006 and 2007, 279 school children in the seventh grade across eastern Norway completed a survey assessing their GSE and HRQOL. The children were from schools that had been randomly selected using cluster sampling. T-tests were computed to compare mean subscale values between HRQOL and socio-demographic variables. Single and multiple regression analyses were performed to explore associations among GSE, HRQOL and socio-demographic variables.

**Results:**

Regression analyses showed a significant relationship between increasing degrees of GSE and increasing degrees of HRQOL. In analyses adjusted for socio-demographic variables, boys scored higher than girls on self-esteem. School children from single-parent families had lower scores on HRQOL than those from two-parent families, and children who had relocated within the last five years had lower scores on HRQOL than those who had not relocated.

**Conclusion:**

The strong relationship between GSE and HRQOL indicates that GSE might be a resource for increasing the HRQOL for school children.

## Background

Health-related quality of life (HRQOL) is a multidimensional construct that consists of physiological, psychological and functional aspects of well-being as seen from the individual's own perspective [1]. HRQOL can be used as an outcome measure of school children's well-being, and for developing methods to promote health [2]. The concept of health promotion comprises active support of the physical, social and mental well-being of individuals [3,4]. Schools are important settings for health promotion for children [5,6]. Research has thus far mainly focused on symptoms and problems [7,8]. Therefore, more research on HRQOL and psychosocial factors that may enhance the well-being of school children is needed. The concept of self-efficacy is suggested as one such focus. Introduced by Albert Bandura, it represents one core aspect of his social cognitive theory [9]. Self-efficacy comprises both general and domain-specific measures. General self-efficacy (GSE) is the belief in one's competence to attempt difficult or novel tasks, and to cope with adversity arising from specific demanding situations [10-12]. It makes a difference to how people feel, think and act [9]. The construct of GSE reflects an optimistic self-belief [13], and refers to a global confidence in coping abilities across a wide range of demanding situations [13].

According to social cognitive theory [9], human motivations and actions are regulated extensively by forethought. The prime factor for influencing behaviour is perceived self-efficacy [11]. Self-efficacy is the foundation of human motivation, well-being and accomplishment. Perceived self-efficacy can be characterized as being competence based, prospective, and action related [9].

According to Bandura [9], self-efficacy is context dependent and assessment methods must be tailored to each event or research setting. However, other researchers have proposed the concept of general self-efficacy and have constructed a general self-efficacy scale for use in several settings [14]. The essential idea behind this concept is that self-efficacy can be of a general character or a universal construct [15], and can be used in a wide range of situations.

Previous studies have found that a high degree of GSE is related to high self-belief [9,16] and an optimistic outlook on life [13,17]. An overview of the literature shows that positive associations between GSE and HRQOL [12,18,19] as well as domain-specific self-efficacy [10,20-23] were found among adults with diseases. Only one study among adolescents focused on associations between self-efficacy and life satisfaction. While they are superficially similar, life satisfaction and HRQOL are not the same concept [24]. This study found a positive association between the domain-specific self-efficacy concept (measured as family self-efficacy and peer self-efficacy) and perceived life satisfaction (measured by variables such as family life, friends, school, community, financial status, and material possessions). Additionally, a relatively new study [25] has found that stress-related coping was a significant predictor for quality of life among children with asthma.

In addition to the direct and positive association between self-efficacy and different health outcomes, Bandura [9] has suggested that self-efficacy might function as a mediator between stress experience and negative health and well-being outcomes.

No previous studies have explored associations between GSE and HRQOL in healthy school children. The main aim of this study was to examine the association between GSE and HRQOL in a sample of Norwegian school children, and explore how this association is related to socio-demographic characteristics. Based on both empirical research and theory, we hypothesized that increasing degrees of GSE would be related to increasing degrees of HRQOL.

## Methods

### Sample

This study was part of a larger study that had the overall aim of studying HRQOL among Norwegian school children and adolescents aged 8-18 years. Data collection was carried out from October 2006 to April 2007. The school children were recruited through schools in a region of eastern Norway containing about 1.7 million inhabitants (36% of the total Norwegian population). Statistics Norway drew a cluster sample of 11 randomly selected primary schools using the following criteria: geographic spread, rural and urban districts, small and large schools. The schools were sent a letter of invitation outlining the study, and were followed up by telephone. Schools that declined to participate were replaced by other schools selected according to the same criteria. Children in seventh grade in the selected schools with sufficient competence in the Norwegian language were included in the study.

The sample in this study consisted of 444 eligible school children in seventh grade (age 12-13 years), of whom 279 participated (the response rate was 63%). Eighty-three children (19%) had forgotten to obtain informed consent from parents, 41 children (9%) were absent from school on the day of the study, 30 children (7%) received the wrong questionnaire, and 11 children (2%) declined to participate.

### Procedure

The school children and their teachers were given verbal and written information at school by the investigator one week before the study took place. The children were told that the purpose of the study was to obtain knowledge about general quality of life among children and adolescents. They were also informed that their responses would be treated anonymously, and that there were no right or wrong answers. The children received an envelope with standard information about the study, and written consent form for their parents. Approvals signed by parents and children were returned to the teachers. The school nurse assisted in the information gathering process and in the data collection tasks. The self-report instruments were completed in the classrooms during a school hour, and the investigator was present and could assist the children if necessary. Children who were absent from school on the day of the study were not included.

### Measures

#### HRQOL

The Norwegian translation of the German questionnaire KINDL was used to measure HRQOL. KINDL is a quality of life measure developed for use with healthy and clinical groups of children and adolescents aged 4-16 years. The questionnaire has been developed as a generic measure. However, some disease-specific modules are available and can be added to the generic measure. Only the generic instrument was used in the present study. The measurement is easy to use, and suitable for use in school health services. The form consists of 24 Likert-scaled items equally divided into six subgroups (physical well-being, emotional well-being, self-esteem, family, friends and school). Each item refers to experiences over the past week and is rated on a five-point scale (1 = Never, 2 = Seldom, 3 = Sometimes, 4 = Often and 5 = Always). Mean scores are calculated for each of the six subscales and for the total scale, and linearly transformed to a 0-100 scale.

KINDL has satisfactory reliability and validity, and its psychometric properties have been tested in several countries including Norway [3]. Cronbach's α was from 0.53 to 0.78 for the subscales, and 0.82 for the total scales in the Norwegian study [3], and 0.70 and higher for the subscales and 0.80 for the total scale in other studies [1,26]. Correlations with comparable well-being scales have shown acceptable convergent validity and a high correlation (r >.70) with subscales of the Child Health Questionnaire [27] as well as satisfactory discriminant validity [1].

#### GSE

GSE refers to global confidence in one's ability across a wide range of demanding and novel situations [14]. The Generalized Self-Efficacy Scale is a 10-item psychometric scale that is designed to assess optimistic self-belief in coping with a variety of difficult demands in life. The scale was originally developed in Germany by *Matthias Jerusalem *and *Ralf Schwarzer *in 1981 and has been used in many studies with hundreds of thousands of participants [14]. The scale was created to assess a general sense of perceived self-efficacy, with the aim in mind of predicting ability to cope with daily demands as well as adaptation after experiencing all kinds of stressful life events. A revised five-item version of this instrument was used in the present study [28,29]. The scale is designed for the general adult population, including adolescents from 12 years old. A typical item was, "I always manage to solve difficult problems if I try hard enough." The instrument has a four-point scale from 1 ("completely wrong") to 4 ("completely right"). Higher scores refer to higher levels of GSE.

The GSE scale has been found to be reliable and valid in numerous studies, where the Cronbach's α was between 0.75 and 0.90 [14]. It has also proved valid in terms of convergent and discriminate validity. It correlates positively with self-esteem and optimism [14]. Criterion-related validity is documented in numerous correlation studies where positive coefficients were found with favourable emotions, dispositional optimism, and work satisfaction. Negative coefficients were found with depression, anxiety, stress, burnout, and health complaints [14].

### Ethics

The Regional Committee for Medical Research Ethics for Western Norway approved the study. Written informed consent for the participation was obtained from the parents and the children before they could complete the questionnaires. The children were informed that their responses would be treated anonymously, and that there were no right or wrong answers.

### Statistical analysis

Descriptive analyses were used to assess the mean and standard deviation of HRQOL (subscales and total scale) for socio-demographic variables and GSE (total). Cronbach's alpha was computed to assess the reliability of the questions. T-tests were done to compare mean subscale values of HRQOL according to groups of socio-demographic variables. Sociodemographic variables that showed significant differences for any subscale were included in the regression analyses. To evaluate the associations between HRQOL as a dependent variable, socio-demographic variables, and GSE as an independent variable, single and multiple regression analyses were performed.

Regression analyses were performed to evaluate the association between HRQOL, and sociodemographic variables and GSE. Both single and multiple regression analyses were performed. In the multiple models, we included HRQOL as a dependent variable, and gender (girls versus boys), marital status (two parents married or cohabiting) versus single parent (unmarried, divorced or widowed), relocation in the last five years (yes versus no), mother's birthplace (Norway versus other country), and GSE as independent variables.

To test for heterogeneity with gender in analyses of relationships between GSE and HRQOL, an interaction term was included in the statistical model.

According to the manual, the missing values of HRQOL and GSE were imputed with the mean of the non-missing items if the respondent had answered at least 70% of the items in the actual subscale. HRQOL and GSE were transformed on a scale from 0 to 100. GSE was analysed as a total score. The p-value of this interaction term was not significant, and the regression analyses were therefore performed with both genders combined. A p-value less than or equal to 0.05 was considered statistically significant. All analyses were conducted using SPSS Version 15 for Windows (SPSS Inc., Chicago, Illinois).

## Results

### Mean scores and Cronbach's alpha

As seen in Table 1, a total of 279 school children answered the questionnaire; 152 (55%) girls and 127 (45%) boys, all in seventh grade. The school children in the present study were 12-13 years old. Results from all the subscales and total scale in HRQOL are presented in Table 2. The total mean score for HRQOL was 72.6, and the total mean score for GSE was 67.7; by gender, 66.3 for girls and 69.4 for boys. Reliability is expressed by Cronbach's α, where the overall value for HRQOL was 0.82. In the present study, the internal consistency of the Norwegian KINDL friends and school subscale showed the lowest alpha, while the self-esteem subscale showed the highest values. Cronbach's α for GSE was 0.79.

**Table 1:** Sociodemographic characteristics of seventh-grade school children (n = 279)

**Characteristic**	**n**	**%**
Gender		
Girls	152	55
Boys	127	45
Marital status		
Two parents	192	70
Single parent	84	30
Unknown	3	
Mother's birthplace		
Norway	235	84
Other country	40	14
Unknown	6	2
Father's birthplace		
Norway	232	83
Other country	42	15
Unknown	5	2
Relocated in last 5 years		
No	182	66
Yes	94	34
Unknown	3	

**Table 2:** Subscale and total scale of Health-Related Quality of Life (HRQOL) and General Self-Efficacy (GSE) among seventh-grade schoolchildren (n = 279)

	**n**	**Mean**	**Standard deviation**	**No. items**	**Cronbach's alpha**
HRQOL					
Physical wellbeing	273	72.56	17.56	4	0.70
Emotional wellbeing	275	78.54	15.19	4	0.67
Self-esteem	275	62.37	19.43	4	0.79
Family	275	77.92	17.38	4	0.76
Friends	274	74.45	15.18	4	0.61
School	273	70.03	16.93	4	0.61
Total scale	265	72.66	12.38	24	0.82
					
GSE	275	67.70	19.69	5	0.79

All the scales are transformed to 0--100.

### Socio-demographic variables

Additional file 1 shows mean values for the subscales of HRQOL according to sociodemographic variables. The only significant difference between boys and girls was for self-esteem, where boys reported higher scores than girls. The marital status variable showed that children with a single parent had lower scores on all subscales and totals compared to those with two parents. Respondents who had relocated in the previous five years had lower scores on the subscales and total HRQOL. Those children whose mothers came from a country other than Norway had significantly higher scores on the subscales for self-esteem and family. The highest mean score was on the subscale emotional well-being for respondents with two parents (80.4), while the lowest score was observed for self-esteem for respondents with single parents (56.7).

### Regression analyses of socio-demographic variables, GSE and HRQOL

Results from single and multiple regression analyses of socio-demographic variables, GSE and HRQOL are presented in Additional files 2 and 3. The findings from regression analyses show that boys had significantly higher scores on self-esteem than girls. Respondents with a single parent had negative coefficients in all the subscales, and significantly lower scores on emotional well-being and total HRQOL score compared with those with two parents. School children who reported having relocated in the last five years had negative coefficients in all the subscales, and significantly lower scores on family compared with those who had not relocated. Participants whose mothers were born in a country other than Norway had significantly higher scores on the subscales for self-esteem and family on HRQOL compared with children with a Norwegian mother. No significant differences were found for the school children's fathers' country of origin.

Results from linear regression analyses of GSE and HRQOL showed that increasing degrees of GSE are significantly related to an increasing degree of HRQOL in all analyses, both adjusted and unadjusted. The strongest association for GSE is the subscale for self-esteem and the school score.

## Discussion

The main finding from the present study is that GSE was significantly and positively associated with HRQOL in healthy school children. An increasing degree of GSE was related to an increasing degree of HRQOL for all subscales and total scales of HRQOL. This result was consistent for both boys and girls. The strongest association was for self-esteem, but even physical well-being was significantly, albeit weakly associated. Respondents with a single parent had lower scores on the emotional well-being subscale and the total HRQOL score compared with those who had two parents.

This research on HRQOL was based on school children's subjective perspective, and refers to individual internal judgments about quality of life experience as opposed to problems, symptoms or diagnoses. HRQOL is a positive phenomenon in which the school children report their life satisfaction. This phenomenon is relative, and can be influenced by individual needs and expectations [2]. The present study found almost the same value in the subscales of HRQOL as a prior study in Norway [3,30,31]. The highest mean score was for emotional well-being, followed by friends and family, and the lowest score was for self-esteem [3,30,31].

As in previous research [30-33], the present study found that boys reported higher scores in the self-esteem subscale than girls. Other studies that have explored gender differences on self-esteem with other instruments (Rosenberg Self-esteem scale and Harter Self-esteem scale) have shown that girls had lower self-esteem than boys [33,34]. Self-efficacy and self-esteem have shown similarities. Luszynska et al. [11] found that optimism, self-regulation and self-esteem had the highest positive association with self-efficacy [11]. Self-esteem refers to a conviction about one's worth, whereas the concept of self-efficacy pertains to judgments of one's personal ability to act [9].

School children who reported that they lived with a single parent had a lower score on HRQOL in emotional well-being and total HRQOL than those who lived with two parents. Other studies on quality of life [35] and life satisfaction [24] have showed that children living with a single parent had lower scores on quality of life than those living with two parents.

As with earlier research on adults [10,13,18,22], the strongest positive association in the present study was between GSE and HRQOL. Leganger et al. [36] found significant positive correlations between GSE scale and life satisfaction among adults. Previous studies have found that GSE predicts health outcomes [11,37,38], happiness [16], optimism, hope and well-being [17]. A strong sense of GSE was also related to higher achievement and better social integration [9,15]. One study explored the relationships between GSE and well-being among adults in five different countries, and found evidence for positive associations between GSE and quality of life and self-esteem [11]. The only previous study that has explored the relationship between life satisfaction and self-efficacy among school children found that self-efficacy beliefs were related to overall life satisfaction [24].

It was interesting that even physical well-being was positively correlated with GSE, because physical well-being is a statement of how the school children reported their health status, while GSE is a theoretical concept built on their belief in themselves and their level of optimism. Other studies among adolescents have found associations between low physical activity and low self-efficacy [39-41].

Bandura's social cognitive theory is based on an understanding that humans are direct agents in shaping and responding to environmental conditions. A strong sense of personal self-efficacy is related to better health [9,15]. The level of self-efficacy varies by age, personal experiences, and differs individually. Pubertal changes contribute to the development of self-efficacy in interaction with psychosocial factors. Adolescents must re-establish their sense of efficacy, social connectedness and network of new peers and with multiple teachers. During this period adolescents become less confident [9]. A person who believes in being able to produce a desired effect can lead a more active and self-determined life. The belief of 'can do' cognition is a sense of control over one's environment [12]. A high level of self-efficacy is related to positive emotions and effective problem solving [9]. High self-efficacy beliefs are also related to life satisfaction. Therefore, quality of life is high in self-efficacious individuals [11]. Cicerone et al. [20] found that perceived GSE was strongly associated with life satisfaction among adults.

Self-efficacy is a concept that can possibly change, according to Bandura [9]. GSE is a characteristic that can be altered through education programming [10,37]. An optimistic self-belief helps in setting goals, initiating actions and maintaining motivation [13]. People with a high level of self-efficacy choose to perform more challenging tasks. They set themselves higher goals and stick to them [9]. School settings are areas with potential for changes that can improve school children's health, well-being and self-efficacy. School staff and health professionals can help school children set realistic goals with tasks that they are able to manage, so they can learn from earlier positive experiences and expect to master tasks in the future. Self-efficacy and the feeling of being able to achieve certain goals using one's capacities play fundamental roles in the health and well-being of school children [9].

### Strengths and limitations

Several limitations of this study should be considered when interpreting the results. The sample size was quite small, which restricts the number of factors included in the multivariate testing. The response rate was 63%. Another limitation is that we have no information about the school children who did not participate in this study. We cannot assess whether participants and non-participants differed in any respect. As the study had a cross-sectional design, we cannot draw any strong practical implications from it. Moreover, in view of this design, we can only interpret the results as associations. Although the applied regression model implicitely defines GSE as an explanatory factor for HRQOL, a bidirectional effect is possible. An increase in GSE might result in better HRQOL, or school children who have better HRQOL might also score higher on GSE. However, our findings were consistent with previous theoretical and empirical work. Bandura [9] also suggested that self-efficacy predicts better health and well-being.

Further research that uses longitudinal research, randomized control trials (RCT) or other designs is needed to determine causality. Longitudinal research could examine this relationship more closely and determine the direction of the associations found in this study. RCT could be used in intervention studies to examine whether GSE might be a predictor of HRQOL for school children. Confounding by other factors could be a potential problem, in addition to the general problem of verifying causal relationships. However, the strong significant relationship between GSE and HRQOL indicates that the observed associations could not be completely explained by other factors. A strength of this study was the randomly selected sample. The sample was drawn from different schools in eastern Norway. The school children were all in the seventh grade. The Norwegian school system is rather homogenous, so the findings should be similar in other Norwegian populations in the same age group. Findings from this study and previous research indicate that the school setting could have the potential for changes that can improve HRQOL for school children.

## Conclusion

Results from this study showed a strongly positive significant association between GSE and HRQOL. Assessing HRQOL among school children enables school health services to determine their life conditions, discover threats to their well-being, and become aware of vulnerable school children. The hypothesis that we will find positive relationships between GSE and HRQOL among school children was confirmed.

School settings are areas with a potential for changes that can improve school children's self-efficacy and health. The school is important for children's social and emotional development. Thus, intervention strategies that are aimed at improving self-efficacy and HRQOL are needed in schools. More research is needed to determine whether the school health service should implement interventions such as discussion groups that aim to help school children to reach their goals and strengthen their self-efficacy, with support from school staff, health professionals, family and peers.

## Abbreviations

GSE: General Self-Efficacy; HRQOL: Health-Related Quality of Life; KINDL: Kinder Lebensqualität Fragebogen (German Language Questionnaire for Measuring Health-Related Quality of Life in Children and Adolescents); SPSS: Statistical Software Package for the Social Sciences; WHO: World Health Organization.

## Competing interests

The authors declare that they have no competing interests.

## Authors' contributions

LGK contributed to the study design, data collection, statistical analysis, interpretation of data and drafting of the paper. KH contributed to data collection and revision of the manuscript. SH contributed to the study design, statistical analysis, interpretation of data and revision of the manuscript. RS contributed to statistical analysis, interpretation of data and revision of the manuscript. GKN contributed to the study design, statistical analysis, interpretation of data and revision of the manuscript. All authors read and approved the final manuscript.

## Supplementary Material

Additional file 1 **Health-Related Quality of Life (HRQOL) according to sociodemographic variables (n = 279)**. The data provided represent the statistical analysis of t-tests to compare mean subscales value of HRQOL according to groups of socio-demographic variables. Click here for file

Additional file 2 **Regression coefficients (Reg. coeff.) with 95% confidence interval (CI) and standardized coefficients (Stand. coeff.) for linear association of subscore of health-related quality of life (HRQOL), socio-demographic variables and general self-efficacy (GSE)**. The data provided represent the statistical analysis to evaluate the associations between HRQOL, and socio-demographic variables and GSE. Single and multiple regression analysis were performed. (n = 279). Click here for file

Additional file 3 **Regression coefficients (Reg. coeff.) with 95% confidence interval (CI) and standardized coefficients (Stand. coeff.) for linear association of subscore of health-related quality of life (HRQOL), socio-demographic variables and general self-efficacy (GSE)**. The data provided represent the statistical analysis to evaluate the associations between HRQOL, and socio-demographic variables and GSE. Single and multiple regression analysis were performed. (n = 279). Click here for file
