# Supplementary material for: Associations between general self-efficacy and health-related quality of life among 12-13-year-old school children: a cross-sectional survey
Source: Health Qual Life Outcomes. 2009 Sep 23;7:85. doi: 10.1186/1477-7525-7-85 (PMC2757020; doi:10.1186/1477-7525-7-85)
Supplement: Additional file 1 — Health-Related Quality of Life (HRQOL) according to sociodemographic variables (n = 279). The data provided represent the statistical analysis of t-tests to compare mean subscales value of HRQOL according to groups of socio-demographic variables. [file 1477-7525-7-85-S1.DOCX]

### Table 3 Health-Related Quality of Life (HRQOL) according to sociodemographic variables (n=279)

|  | **1. Physical well-being** Mean SD** | **2. Emotional well-being**  **Mean SD**** | **3. Self-esteem**  **Mean SD**** | **4. Family**  **Mean SD**** | **5. Friends**  **Mean SD**** | **6. School**  **Mean SD**** | **7. Total**  **Mean SD**** |
| --- | --- | --- | --- | --- | --- | --- | --- |
| Gender  Girls  Boys P* Marital status  Two parents  Single parent P* Relocated last year  No  Yes P* Mother’s birthplace  Norway  Other country P* | 70.90 (18.41)  74.92 (16.15)  0.06  74.21 (16.50)  69.43 (19.40)  **0.03**  73.81 (18.01)  70.70 (16.53)  0.16  73.14 (17.14)  70.23 (20.10)  0.34 | 78.34 (15.37)  78.59 (15.15)  0.89  80.39 (14.18)  73.95 (16.80)  **<0.01**  79.73 (13.95)  75.92 (17.38)  **0.05**  78.45 (15.08)  79.01 (16.19)  0.83 | 59.47 (17.89)  65.56 (20.50)  **<0.01**  64.84 (19.53)  56.70 (18.06)  **<0.01**  64.09 (19.30)  58.98 (19.32)  **0.03**  61.38 (19.11)  68.91 (19.63)  **0.02** | 77.26 (18.35)  78.83 (15.41)  0.45  79.87 (16.54)  73.32 (18.49)  **0.01**  79.62 (17.00)  74.56 (17.71)  **0.02**  76.91 (17.65)  83.97 (14.53)  **0.01** | 74.99 (14.72)  73.28 (15.48)  0.35  75.12 (13.77)  71.71 (17.51)  0.08  74.20 (14.89)  73.84 (15.48)  0.85  74.22 (14.31)  73.18 (19.17)  0.69 | 69.07 (16.96)  70.90 (17.00)  0.37  71.85 (16.41)  65.19 (17.50)  **<0.01**  71.14 (16.06)  67.26 (18.53)  0.07  69.77 (17.07)  70.35 (16.94)  0.84 | 71.81 (12.38)  73.65 (12.35)  0.22  74.36 (11.86)  68.50 (12.79)  **<0.01**  73.78 (11.73)  70.31 (13.44)  **0.03**  72.37 (12.26)  74.44 (13.27)  0.34 |

*T-test

** Standard deviation of the mean
