# Supplementary material for: Associations between general self-efficacy and health-related quality of life among 12-13-year-old school children: a cross-sectional survey
Source: Health Qual Life Outcomes. 2009 Sep 23;7:85. doi: 10.1186/1477-7525-7-85 (PMC2757020; doi:10.1186/1477-7525-7-85)
Supplement: Additional file 2 — Regression coefficients (Reg. coeff.) with 95% confidence interval (CI) and standardized coefficients (Stand. coeff.) for linear association of subscore of health-related quality of life (HRQOL), socio-demographic variables and general self-efficacy (GSE). The data provided represent the statistical analysis to evaluate the associations between HRQOL, and socio-demographic variables and GSE. Single and multiple regression analysis were performed. (n = 279). [file 1477-7525-7-85-S2.DOC]

**Table 4. Regression coefficients (Reg. coeff.) with 95% confidence interval (CI) and standardized coefficients (Stand. coeff.) for linear association^a^ of subscore of health-related quality of life (HRQOL),^b^ sociodemographic variables^c^ and general self-efficacy (GSE)^b^. Unadjusted and adjusted regression analysis. (n=279)**

|  | **1. Physical well-being**  **Reg. coeff.**  **(95% CI)** | **Stand. coeff.** | **p** | **2. Emotional well-being**  **Reg. coeff.**  **(95% CI)** | **Stand. coeff.** | **p** | **3. Self-esteem**  **Reg. coeff.**  **(95% CI)** | **Stand. coeff.** | **p** |
| --- | --- | --- | --- | --- | --- | --- | --- | --- | --- |
| Gender  Unadjusted  Adjusted  Marital status  Unadjusted  Adjusted  Relocated  Unadjusted Adjusted Mother’s birthplace  Unadjusted Adjusted GSE  Unadjusted  Adjusted  R ² | 4.01 (-0.15-8.17)  2.75 (-1.42-6.94)  -4.78 (-9.31-,-0.25)  -3.12 (-7.99-1.74)  -3.11 (-7.53-1.30)  -0.98 (-5.68-3.70)  -2.91 (-8.97-3.15)  -3.37 (-9.57-2.81)  0.20 (0.09-0.30)  0.18 (0.08-0.29)  0.073 | 0.11  0.07  -0.12  0.08  -0.08  -0.02  -0.05  -0.06  0.22  0.21 | **0.05**  0.19  **0.03**  0.20  0.16  0.67  0.34  0.28  **<0.01**  **<0.01** | 0.24 (-3.39-3.88)  -0.86 (-4.40-2.67)  -6.44 (-10.34-,-2.54)  -4.56 (-8.69--0.44)  -3.80 (-7.63-0.02)  -2.35 (-6.34-1.63)  0.55 (-4.63-5.75)  0.53 (-4.66-5.73)  0.23 (0.14-0.32)  0.20 (0.11-0.29)  0.116 | 0.01  -0.02  -0.19  -0.13  -0.11  -0.07  0.01  0.01  0.29  0.26 | 0.89  0.63  **<0.01**  **0.03**  **0.05**  0.24  0.83  0.83  **<0.01**  **<0.01** | 6.09 (1.53-10.65)  4.06 (-0.08-8.20)  -8.14 (-13.07-,-3.20)  -3.17 (-8.00-1.65)  -5.11 (-9.96—0.26)   -3.97 (-8.63-0.69)  7.53 (0.99-14.07)  6.01 (-0.07-12.01)  0.44 (0.33-0.54)  0.41 (0.30-0.51)  0.247 | 0.15  0.10  -0.19  -0.07  -0.12  0.09  0.13  0.10  0.44  0.41 | **0.01**  **0.05**  **<0.01**  0.19  **0.03**  0.09  **0.03**  **0.05**  **<0.01**  **<0.01** |

**ª** Multiple linear regression analysis adjusted for all the other remaining variables

**^b^** Continuous scale ranging from lowest degree to highest degree. Scores are transformed to 0-100

**^c^** Dichotomized variables: Gender (0=girls, 1=boys). Marital status (0=two parents, 1=one parent) Relocated in last 5 years (0=No, 1=Yes), Mother’s birthplace (0= born in Norway, 1= born in other country)
