# Supplementary material for: Associations between general self-efficacy and health-related quality of life among 12-13-year-old school children: a cross-sectional survey
Source: Health Qual Life Outcomes. 2009 Sep 23;7:85. doi: 10.1186/1477-7525-7-85 (PMC2757020; doi:10.1186/1477-7525-7-85)
Supplement: Additional file 3 — Regression coefficients (Reg. coeff.) with 95% confidence interval (CI) and standardized coefficients (Stand. coeff.) for linear association of subscore of health-related quality of life (HRQOL), socio-demographic variables and general self-efficacy (GSE). The data provided represent the statistical analysis to evaluate the associations between HRQOL, and socio-demographic variables and GSE. Single and multiple regression analysis were performed. (n = 279). [file 1477-7525-7-85-S3.DOC]

**Table 5. Regression coefficients (Reg. coeff.) with 95% confidence interval (CI) and standardized coefficients (Stand. coeff.) for linear association^a^ of subscore of health-related quality of life (HRQOL),^b^ sociodemographic variables^c^ and general self-efficacy (GSE)^b^. Unadjusted and adjusted regression analysis. (n=279)**

|  | **4. Family**  **Reg. coeff.**  **(95% CI)** | **Stand. coeff.** | **p** | **5. Friends**  **Reg. coeff.**  **(95% CI)** | **Stand. coeff.** | **p** | **6.School**  **Reg. coeff.**  **(95% CI)** | **Stand. coeff** | **p** | **7. Total**  **Reg. coeff.**  **(95% CI)** | **Stand. coeff.** | **p** |
| --- | --- | --- | --- | --- | --- | --- | --- | --- | --- | --- | --- | --- |
| Gender  Unadjusted  Adjusted  Marital status  Unadjusted  Adjusted  Relocated  Unadjusted Adjusted Mother’s birthplace  Unadjusted Adjusted GSE  Unadjusted  Adjusted  R ² | 1.56 (-2.57-5.70)  2.07 (-4.04-4.04)  -6.55 (-11.00--2.10)  -4.00 (-8.73-0.71)  -5.06 (-9.39-0.73)  -4.40 (-8.96-0.15)  7.06 (1.18-12.94)  8.08 (2.15-14.01)  0.20 (0.10-0.31)  0.17 (0.07-0.27)  0.109 | 0.04  0.00  -0.17  -0.10  -0.13  -0.12  0.14  0.15  0.23  0.19 | 0.45  1.00  **0.04**  0.09  **0.02**  **0.05**  **0.01**  **0.01**  **<0.01**  **<0.01** | -1.70 (-5.30-1.89)  -2.60 (-6.12-0.92)  -3.41 (-7.30-0.48)  -2.63 (-6.75-1.49)  -0.35 (-4.16-3.46)  0.78 (-3.21-4.78)  -1.04 (-6.19-4.11)  -1.52 (-6.71-3.65)  0.23 (0.15-0.32)  0.23 (0.14-0.32)  0.107 | -0.05  -0.08  -0.10  -0.08  -0.01  0.02  -0.02  -0.03  0.31  0.30 | 0.35  0.14  0.08  0.21  0.85  0.69  0.69  0.56  **<0.01**  **<0.01** | 1.82 (-2.23-5.88)  0.11 (-3.76-3.98)  -6.66 (-11.02-,-2.29)  -4.16 (-8.68-0.35)  -3.87 (-8.16-0.41)  -1.69 (-6.07-2.68)  0.57 (-5.23-6.39)  -1.07 (-6.74-4.60)  0.32 (0.23-0.42)  0.30 (0.21-0.40)  0.162 | 0.05  0.00  -0.18  -0.11  -0.10  -0.04  0.12  -0.02  0.38  0.35 | 0.37  0.95  **0.01**  0.07  0.07  0.44  0.84  0.71  **<0.01**  **<0.01** | 1.84 (-1.15-4.84)  0.37 (-2.38-3.12)  -5.86 (-9.09-2.62)  -3.52 (-6.74-,-0.29)  -3.47 (-6.65-,-0.29)  -2.02 (-5.13-1.07)  2.07 (-2.21-6.36)  1.59 (-2.44-5.63)  0.27 (0.21-0.34)  0.26 (0.18-0.33)  0.224 | 0.07  0.01  -0.21  -0.12  -0.13  -0.07  0.05  0.44  0.44  0.41 | 0.22  0.79  **0.01**  **0.03**  **0.03**  0.20  0.34  0.43  **<0.01**  **<0.01** |

**ª** Multiple linear regression analysis are adjusted for all the other remaining variables

**^b^** Continuous scale ranging from lowest degree to highest degree. Scores are transformed to 0-100

**^c^** Dichotomized variables: Gender (0=girls, 1=boys). Marital status (0=two parents, 1=one parent) Relocated in last 5 years (0=No, 1=Yes), Mother’s birthplace (0=born in Norway, 1= born in other country)
